# The complete plastome of *Acalypha australis* (Euphorbiaceae) and its phylogenetic analysis

**DOI:** 10.1080/23802359.2023.2294891

**Published:** 2024-05-17

**Authors:** Hongqin Li, Changhao Ma, Liqiang Wang

**Affiliations:** a College of Pharmacy, Heze University, Heze, Shandong Province, P. R. China; b Inspection Department Three, Shandong Center for Food and Drug Evaluation and Inspection, Jinan, Shandong Province, P. R. China

**Keywords:** *Acalypha australis*, Euphorbiaceae, plastome, phylogenetic analysis

## Abstract

*Acalypha australis* L. 1753 is a potherb popular among Asian populations and is a traditional herbal medicine. In the current study, the overall genetic diversity of *A. australis* still needs to be better. Here, we assembled and characterized the complete plastome of *A. australis*. The plastome is 168,885 bp in length with a large single-copy (LSC) of 94,576 bp, a small single-copy (SSC) of 19,715 bp, and two copies of inverted repeat region (IRs) of 27,297 bp each. The overall GC content is 34.9%. The plastome contains 127 genes, including 83 protein-coding genes, 36 tRNA genes, and eight rRNA genes. Phylogenomic analysis of the representative species of Euphorbiaceae showed that *A. australis* and *A. hispida* formed a monophyletic sister clade. The results of this study will support further research on the evolution and conservation of the Euphorbiaceae species; they will benefit pharmaceutical applications and ornamentation of the medicinal plant *A. australis*.

## Introduction

*Acalypha australis* L. 1753 is an annual species in the family of Euphorbiaceae, occurring in the subtropical biome. It is a kind of potherb popular among Asian populations, and it is used in traditional Chinese medicine owing to its hemostasis, detoxification, and heat clearance effects.

In previous reports, three active chemical compounds identified from *A. australis* show efficacy in ameliorating ulcerative colitis, including 4-hydroxybenzoic acid methyl ester, 3-methoxy-4-hydroxybenzoic acid, and protocatechuic acid (Li et al. 2007). Acalypha *australis* water-soluble extract has a significant anti-adipogenic effect in the HFD-induced obese mice model (You et al. 2021). In the current study, *A. australis* has been reported to have multiple pharmacological functions, such as anti-inflammatory (Kim et al. 2020) and anti-apoptosis (Shin et al. 2013). However, the overall genetic diversity of *A. australis* still needs to be better understood. In this study, we assembled and characterized the complete plastome of *A. australis* and further performed the phylogenetic analysis of *A. australis*.

## Materials

**Figure 1.**
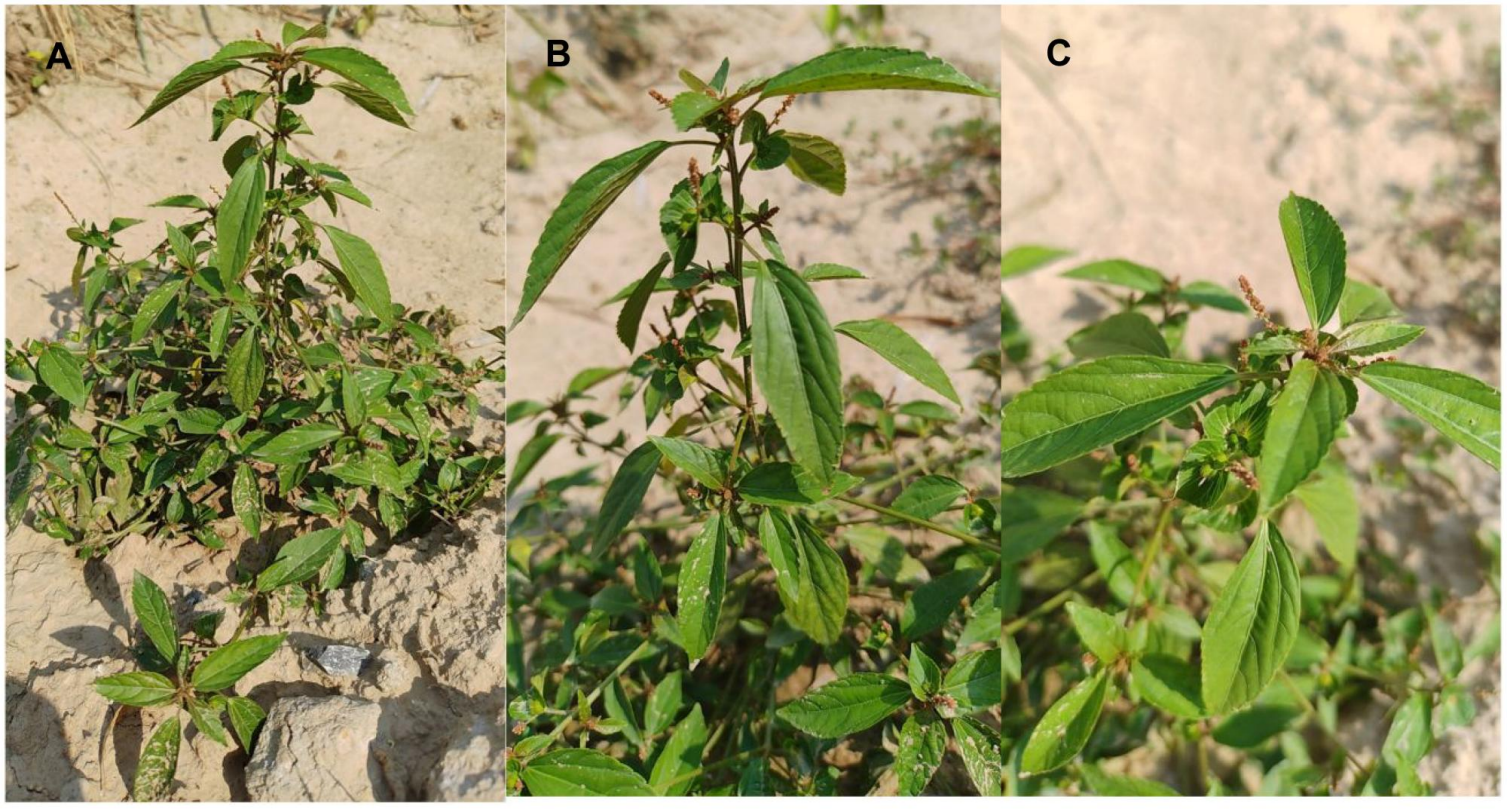
Field photo of *Acalypha australis*. The photo was shot by Liqiang Wang at the position of 35°16′30.0″N, 115°28′41.08″E. Main identifying traits: slender branchlets; leaves membranous, long ovate, subrhomboid ovate or broadly lanceolate; unisexual flowers, inflorescences axillary, thinly terminal; seeds subovate, seed coat smooth. Panel A: Panorama, B: Side profile, C: Bird's-eye view.

The fresh leaves of *A. australis* used for sequencing were obtained from Peony District, Heze City, Shandong Province, China (35°16′30.0″N, 115°28′41.08″E, Figure 1), and a specimen has been deposited in the Heze University Herbarium (contact person: Hongqin Li, machh1111@126.com) under the specimen number HZ20220807. Genomic DNA was extracted using the plant genomic DNA kit (Tiangen Biotech, Beijing, China).

## Methods

The total genomic DNA extract was fragmented into approximately 300 bp short-insert fragments to construct libraries, which were then sequenced using Illumina NovaSeq 6000 technology platforms at Wuhan Benagen Technology Company Limited in Wuhan, China. Trimmomatic 0.35 (Bolger et al. 2014) was used to filter the raw reads by removing adapters and low-quality bases. After filtering, about 16.4 GB of clean reads were obtained and assembled by using GetOrganelle v1.7.1 (Jin et al. 2020). The assembled plastome was annotated using CPGAVAS2 (Shi et al. 2019) and then manually adjusted using Apollo (Pontius 2018). Finally, the annotated plastome was submitted to GenBank with the accession number OQ411035. The circular genome map was obtained from the results of CPGAVAS2 (Shi et al. 2019). We compared the plastomic structure at the LSC/IR and SSC/IR boundary and the homology with another type of species by using IRSCOPE (Amiryousefi et al. 2018) and mVISTA (Frazer et al. 2004) software.

To determine the phylogenetic relationship of *A. australis*, nine other plastomes from the species of Euphorbiaceae were downloaded from GenBank. The species were selected according to the plastome blast results, which showed the closest similarity to that of *A. australis. Brassica rapa* was used as an outgroup. All the eleven plastomes were aligned by using MAFFT software with default parameters (Katoh and Standley 2016), and then a maximum-likelihood (ML) phylogenetic tree was constructed by using IQ-TREE 2.0 (Nguyen et al. 2015) with the Best-fit model of TVM + F+G4 and 1000 bootstrap replicates. To test the reliability of the ML tree, we used the Shimodaira-Hasegawa (SH) (Shimodaira and Hasegawa 1999) and proximately unbiased (AU) (Shimodaira 2002) methods embedded in IQ-TREE (Nguyen et al. 2015). The core test script used was iqtree -s input_mafft.phy -m TVM + F+G4 -z genome.unconstrain.constrain.treels -zb 10000 -zw -au.

## Result and discussion

**Figure 2.**
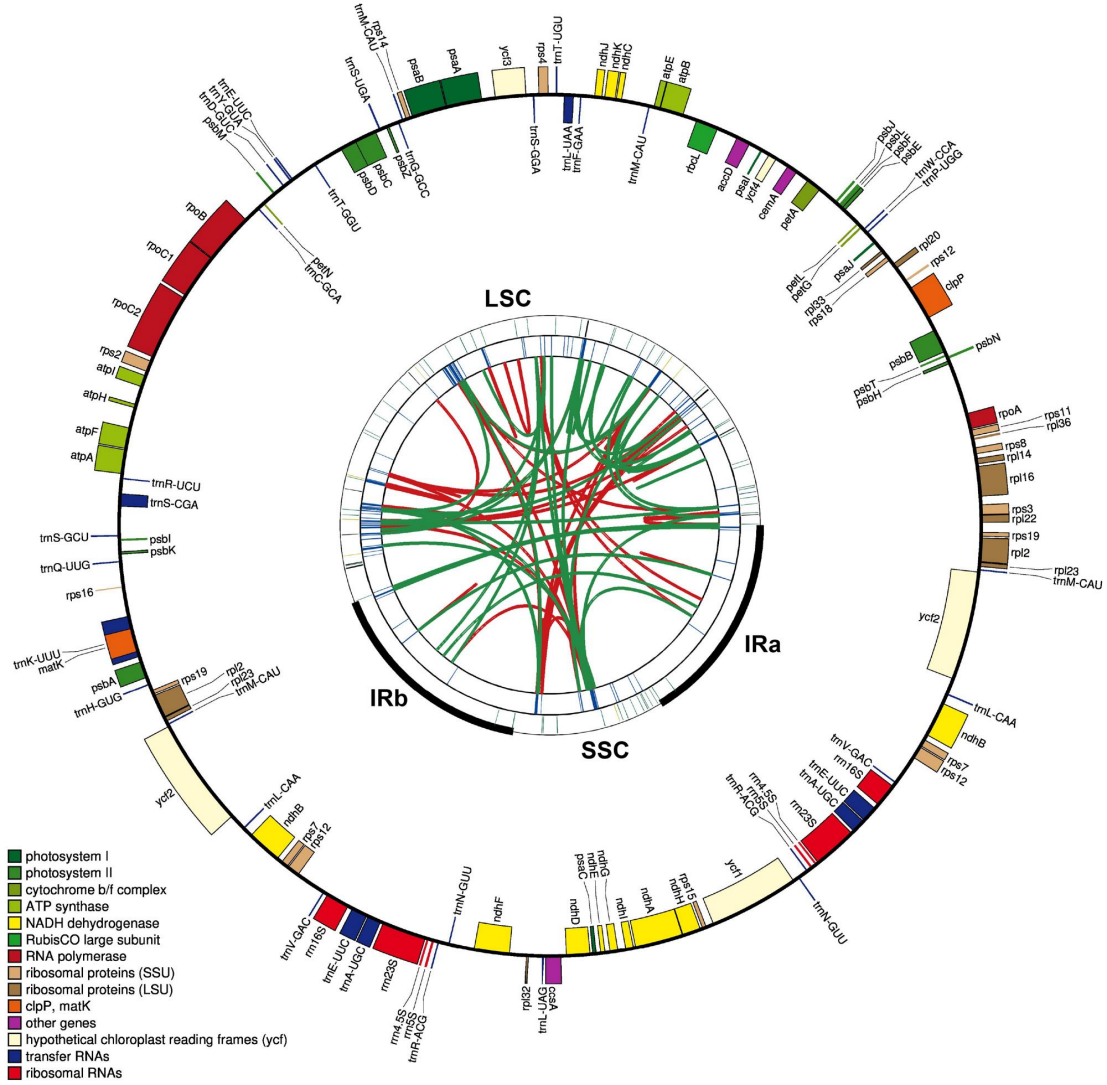
Gene map of the complete chloroplast genome of *Acalypha australis*. The map contains five tracks by default. From the Center outward, the first track shows dispersed repeats, including direct and palindromic repeats connected by red and green arcs. The second track displays long tandem repeats as blue bars, while the third track shows short tandem repeats or microsatellite sequences as differently colored short bars. These colors correspond to the type and description of each repeat, with black representing complex repeats, green for repeat unit size 1, yellow for size 2, purple for size 3, blue for size 4, orange for size 5, and red for size 6. The fourth track displays the SSC, IRa, IRb, and LSC regions. The fifth track shows the genes. The gene names are followed by optional information about codon usage bias and are color-coded based on their functional classification. The inner genes are transcribed clockwise, and the outer genes are transcribed anticlockwise. The functional classification of the genes is shown in the bottom left corner.

The complete plastome of *A. australis* is 168,885 bp long and has a typical quadripartite structure. It consists of two inverted repeat regions (IRs), each of which is 27,297 bp in length, separated by a large single copy region (LSC) of 94,576 bp and a small single copy region (SSC) of 19,715 bp (Figure 2). Mapping experiments showed that the average and minimum coverage depths of the assembled genome were 1911× and 5× (Figure S1). The plastome has a variable GC content distribution; the overall GC content is 34.9%. The GC content of the IR regions is the highest (42.1%), while the corresponding values of the LSC and SSC regions are 32.1% and 28.8%, respectively. Through the analysis of the plastomic structure at the LSC/IR and SSC/IR boundary, the *A. australis* plastome obtained in this study (OQ411035) is 53 bp shorter in overall genome length compared to another *A. australis* plastome (OP477347) already deposited in GenBank. Compared with the deposited plastome, the IR regions in this study are expanded, with the lengths of IRa and IRb each extending by 55 bp. The SSC region also expanded by 9 bp. However, the LSC region was shortened by 172 bp (Figure S2). Due to the expansion of the IR, the *trn*H gene shifted from spanning the LSC/IRb junction to being entirely located within the IRb region; the distance of the *rpl*22 gene from the LSC/IRa junction decreased from 154 bp to 3 bp (Figure S2).

After annotation, 127 genes in the *A. australis* plastome were predicted, including 83 protein-coding genes (PCGs), eight rRNA genes, and 36 tRNA genes. Seven unique PCGs (*rps*19, *rpl*2, *rpl*23, *ycf*2, *ndh*B, *rps*7, and r*ps*12), seven unique tRNA genes (*trn*M-CAU, *trn*L-CAA, *trn*V-GAC, *trn*E-UUC, *trn*A-UGC,*trn*R-ACG, and *trn*N-GUU) and four unique rRNA genes (*rrn*23S, *rrn*16S, *rrn*4.5S, and *rrn*5S) are located at the IR regions. In the whole plastome, six unique PCGs (r*pl*16, *rpo*C1, *atp*F, *rpl*2, *ndh*B, and *ndh*A) contain one intron, 3 PCGs (*ycf*3, *rps*12, and *clp*P) contain two introns (Figure S3A), and five tRNA genes (*trn*K-UUU, *trn*S-CGA, *trn*L-UAA, *trn*E-UUC, and *trn*A-UGC) contain one intron. The *rps*12 was a trans-splicing gene (Figure S3B). The lengths of the protein-coding genes, tRNA genes and rRNA genes in the plastome are 28,083bp, 3,938bp and 9247bp, representing 16.6%, 2.3%, and 5.4% of the total genome length. In the analysis of the plastome homology, the two *A. australis* plastomes show a high degree of similarity, with differences only in some intergenic regions. Among them, the region between the *trn*S-CGA gene and the *trn*S-GCU gene shows the greatest difference (Figure S4).

**Figure 3.**
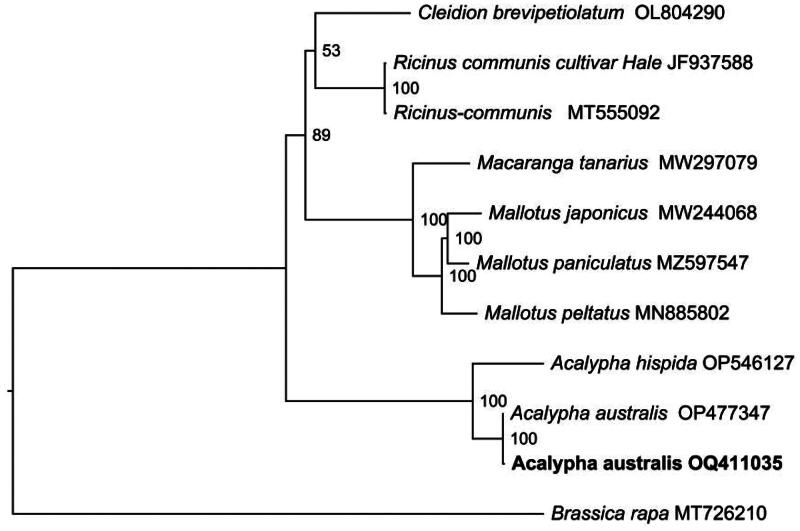
The maximum likelihood phylogeny of *Acalypha australis* and its close relatives using whole genome sequences. The bootstrap values based on 1000 replicates were shown on each node in the cladogram tree. Ten species are from Euphorbiaceae, which are *Mallotus peltatus* (MN885802) (Ke et al. 2020), *Mallotus japonicus* (MW244068) (Wu et al. 2021), *Ricinus communis* (MT555092), *A. hispida* (OP546127), *Mallotus paniculatus* (MZ597547) (Li et al. 2022), *Ricinus communis* (JF937588) (Rivarola et al. 2011), *macaranga tanarius* (MW297079), *cleidion brevipetiolatum* (OL804290), *A. australis* (OP477347) and *A. australis* (OQ411035, in this study). *Brassica rapa* (MT726210) (Wu et al. 2021), from the brassicaceae, served as the outgroup. The new *A. australis* plastomes (OQ411035) in this study were labeled in bold font.

For the phylogenetic analysis of *A. australis* and related taxa within the Euphorbiaceae, we found that two plants of *A. australis* formed a monophyletic clade and had the closest genetic relationship with *A. hispida* (Figure 3). And the reliability of the relationship was strongly supported by 100% bootstrap value. Meanwhile, the topological structure of the ML tree was reliable, with *p*-SH and *p*-AU values of 0.382 and 0.382, respectively. The phylogenetic result in this study will be beneficial to understanding the systematic position of *A. australis* within the Euphorbiaceae.

## Conclusions

This study presents the first characterization of the genome of *A. australis*, which exhibits a typical annular tetrad structure with a size of 168,885 bp and 127 predicted genes. The overall GC content of the genome is 34.9%. In a comparison with another plastome of the same species, it was found that the variation mainly comes from the intergenic regions with less selective pressure, while the length of the IR regions varies to a different extent. The phylogenetic analysis showed that *A. australis* and *A. hispida* formed a monophyletic sister clade. The knowledge of plastome in *A. australis* is beneficial to the exploitation and utilization of the Euphorbiaceae resource and also to the development of conservation genetics and phylogenetic study of the Euphorbiaceae.

## Ethical approval

The *A. australis* is not designated as endangered species. Research on this species requires no specific permissions or licenses.

## Supplementary Material

Supplemental Material

## Data Availability

The complete plastome sequence of *A. australis* in this study has been submitted to the NCBI database (https://www.ncbi.nlm.nih.gov) under the accession number OQ411035. The associated BioProject, Bio-Sample, and SRA numbers are PRJNA928567, SAMN36852646, SRR23328024.
