# Supplementary material for: The complete plastome of Acalypha australis (Euphorbiaceae) and its phylogenetic analysis
Source: Mitochondrial DNA B Resour. 2024 May 17;9(5):636–40. doi: 10.1080/23802359.2023.2294891 (PMC11104693; doi:10.1080/23802359.2023.2294891)
Supplement: Supplemental Material [file TMDN_A_2294891_SM7768.pdf]

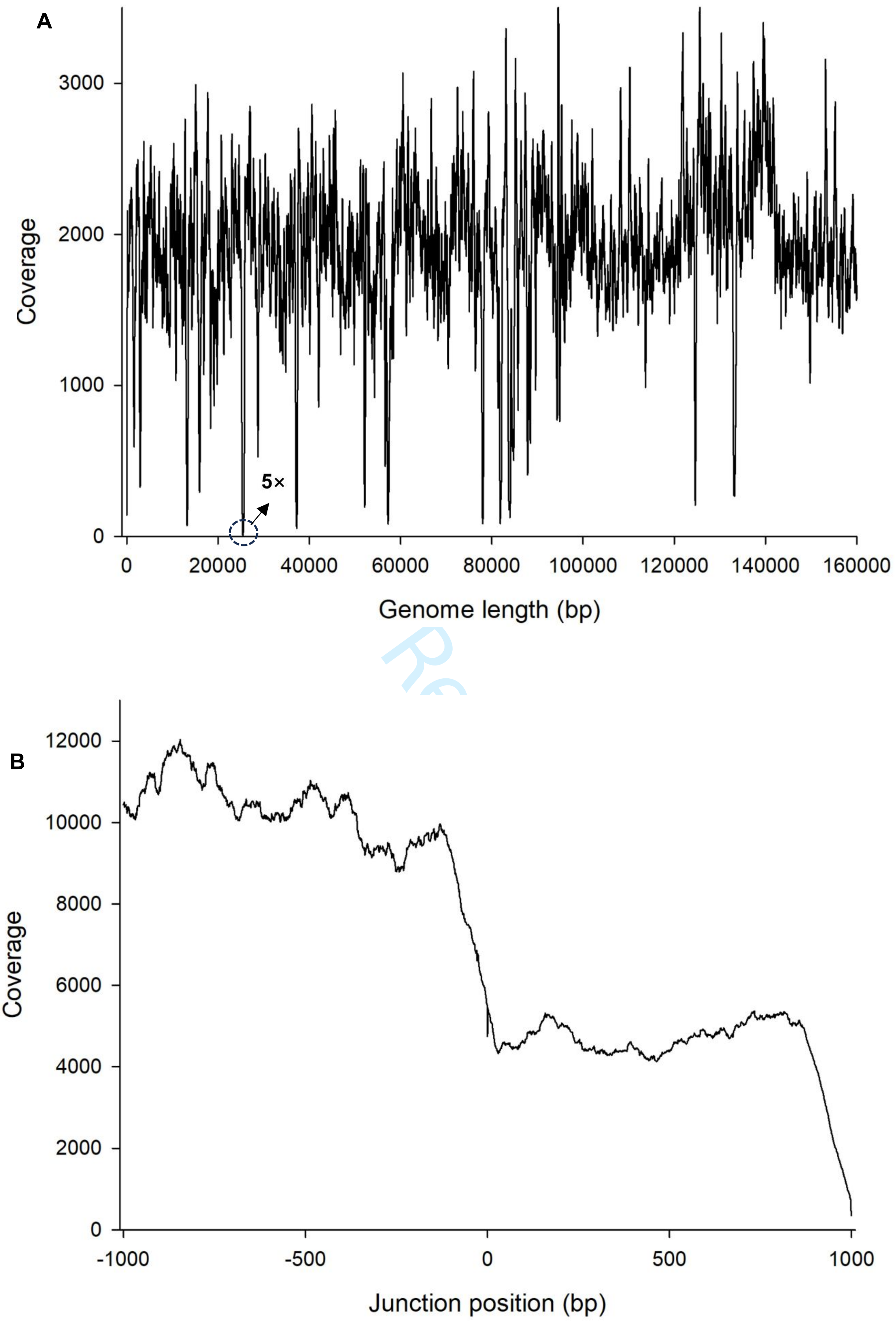
A
Coverage
5×
Genome length (bp)
B
Coverage
Junction position (bp)

C

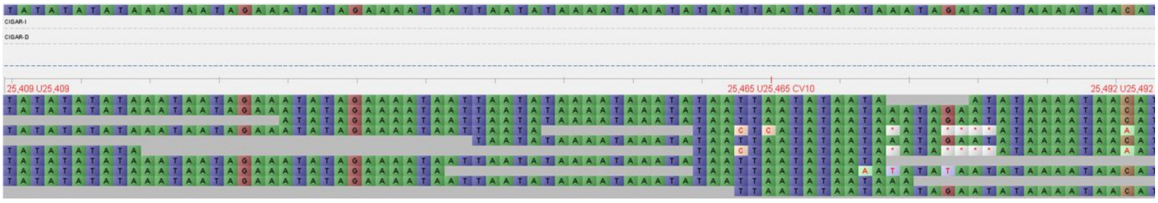

Figure S1. The mapping results of the assembled *Acalypha australis* plastome. (A) The coverage depth of the whole assembled plastome. (B) The coverage depth of the junction position of the plastome. (C) The read coverage of the plastome from position 25,410 to 25,490 with the least coverage depth.

For Peer Review Only

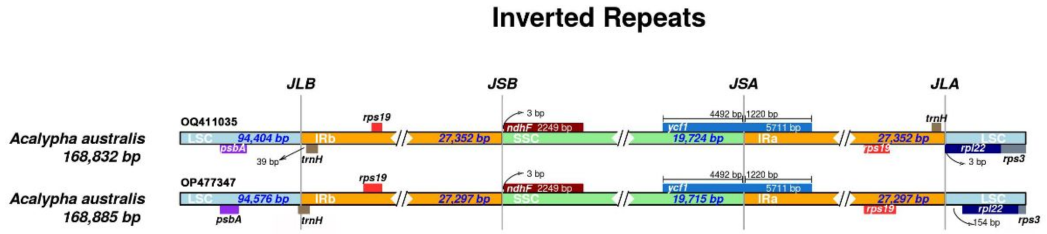

Figure S2. Analysis of the plastomic structure at the LSC/IR and SSC/IR boundary in two types of *Acalypha australis* using IRSCOPE software.

For Peer Review Only

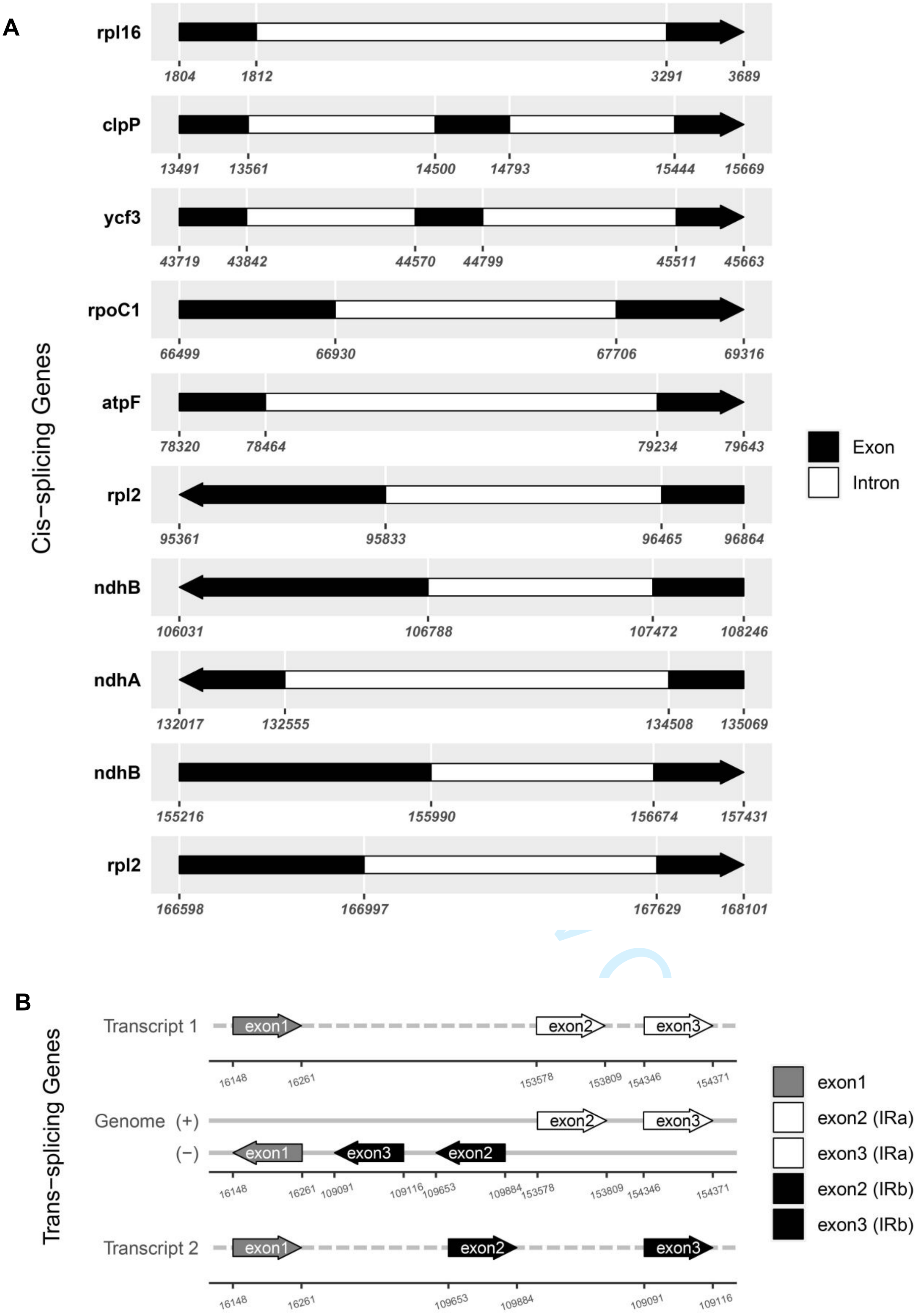

Figure S3. The structures of the genes with introns in the *Acalypha australis* plastome. (A) The structures of 10 cis-splicing genes. (B) The structure of the trans-splicing genes *rps*12.

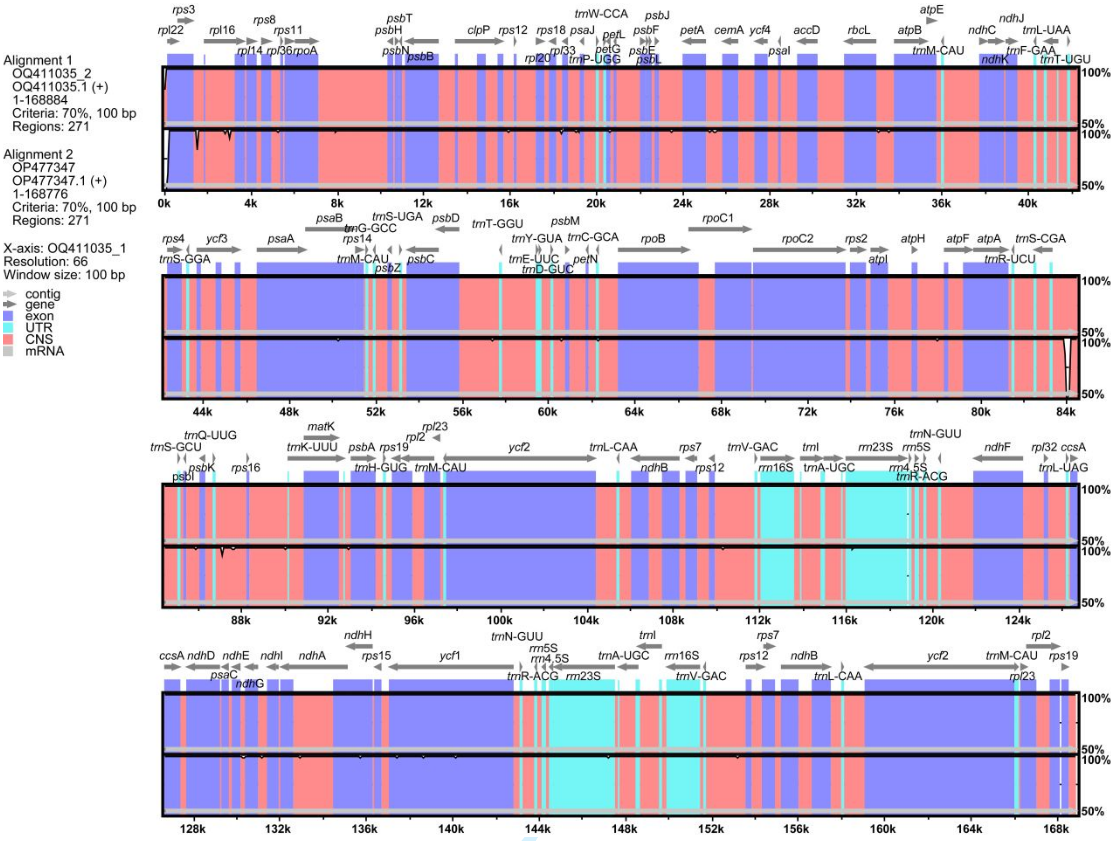

Figure S4. Comparative analysis of homology in the plastome of two types of Amaranthus australis using mVISTA software. The grey arrows indicate the direction of the genes; the Y-axis represents the percentage of similarity (50~100%); the purple bars represent exons, blue bars represent t/rRNA, red bars represent non-coding sequences, and grey bars represent mRNA.

For Peer Review Only
